# Pregnancy and infant outcomes following abatacept exposure during pregnancy

**DOI:** 10.1093/rap/rkag084

**Published:** 2026-07-20

**Authors:** Christina D Chambers, Michelle Ann Caesar, Diana L Johnson, Ronghui Xu, Yunjun Luo, Stephen R Braddock, Margaret P Adam, Luther K Robinson, Kenneth Lyons Jones, OTIS Collaborative Research Group, C Stallman, B Maloney, K Perrotta, A Messer, S Voyer Lavigne, C Senechal, E A Conover, J L Poskochil, P Cole, L Harris-Sagaribay, C Haldeman-Englert, R Muller, N Krstic, R Miller, L Wolfe, S Sherman, K Richardson, M Ashfaq, M Leen-Mitchell, J Carey, C Coles, H Hazard

**Affiliations:** Department of Pediatrics, University of California San Diego, La Jolla, CA, USA; Herbert Wertheim School of Public Health and Human Longevity Science, University of California San Diego, La Jolla, CA, USA; Department of Pediatrics, University of California San Diego, La Jolla, CA, USA; Department of Pediatrics, University of California San Diego, La Jolla, CA, USA; Herbert Wertheim School of Public Health and Human Longevity Science, University of California San Diego, La Jolla, CA, USA; Department of Mathematics, University of California San Diego, La Jolla, CA, USA; Department of Pediatrics, University of California San Diego, La Jolla, CA, USA; Deparment of Pediatrics, Saint Louis University, St. Louis, MO, USA; Department of Pediatrics, University of Washington, Seattle, WA, USA; Department of Pediatrics, State University of New York Buffalo School of Medicine, Buffalo, NY, USA; Department of Pediatrics, University of California San Diego, La Jolla, CA, USA

**Keywords:** abatacept, birth defects, infant outcomes, preterm delivery, pregnancy registry

## Abstract

**Objectives:**

Abatacept is approved for the treatment of moderate–severe RA, JIA and PsA in the USA and elsewhere. The purpose of this study was to estimate the incidence/birth prevalence of selected maternal and infant outcomes in pregnancies exposed to abatacept.

**Methods:**

Pregnant women exposed to any dose of abatacept from the first day of the last menstrual period to the end of the first trimester who resided in the USA or Canada were enrolled in the Organization of Teratology Information Specialists Abatacept Pregnancy Exposure Registry between 2007 and 2019. Data on exposures, outcomes and covariates were collected by maternal interviews, medical records and study-related physical examinations up to 1 year postpartum.

**Results:**

The sample consisted of 30 abatacept-exposed pregnancies. Sixteen were enrolled prospectively and were treated for RA or PsA; 14 did not meet the prospective criteria and were enrolled in an exposure series. Of those prospectively enrolled, 2/13 (15.4%) involved an infant with a major birth defect, 2/16 (12.5%) ended in spontaneous abortion and 2/13 (15.4%) delivered preterm. In the exposure series, 5/14 livebirths (35.7%) ended with an infant with a major birth defect, 2 of which were chromosomal or genetic, and 6/14 (42.9%) delivered preterm.

**Conclusion:**

There were no patterns of major or minor birth defects identified in either group. In the exposure series, selected adverse outcomes were more frequent, likely due to biases that led to exclusion of these pregnancies from the prospective cohort.

Key messages

- No patterns of major or minor birth defects were identified among abatacept-exposed pregnancies.
- Data on abatacept use in pregnancy continues to be very limited.

## Introduction

Abatacept is a medication approved to treat autoimmune disorders, including RA, JIA and PsA, in the USA and elsewhere [1, 2]. The mechanism of action involves preventing T cell activation through binding with CD80 and CD86, thereby blocking the interaction with CD28 [3]. Animal studies performed with doses higher than the standard dose for humans did not result in teratogenic or adverse effects in offspring, however, these results may not be generalizable to humans [4]. There are limited data on the human safety of abatacept when used in pregnancy, including a pharmacovigilance study and a case report [4]. Thus the objective of this study was to estimate the incidence and birth prevalence of selected maternal and infant outcomes in pregnancies exposed to abatacept.

## Methods

MotherToBaby (MTB) pregnancy studies, conducted by the Organization of Teratology Information Specialists (OTIS), are pregnancy registry studies involving participants who reside in the USA and Canada. Counselling services provided by MTB serve to answer questions about the risks and benefits of exposures during pregnancy and are one source of recruitment. Pregnant women who meet the eligibility criteria for a study are referred to the OTIS Research Center at the University of California San Diego (UCSD) where they undergo screening, and if eligible to enrol, are invited to consent to participate. All subsequent data collection occur at the OTIS Research Center.

### Participants

Women exposed to any dose of abatacept from the first day of the last menstrual period (LMP) to the end of the first trimester (with or without continued use in pregnancy) and residing in the USA or Canada were enrolled in the OTIS Abatacept Pregnancy Exposure Registry. Women were divided into the prospective cohort and the exposure series. To qualify for the prospective cohort, women must have had an exposure to at least one dose of abatacept at any time from the first day of the LMP up to and including the 12th week of pregnancy for the treatment of RA or PsA; were currently pregnant and agreed to enrol no later than 20 completed gestational weeks from the LMP; did not have prenatal diagnosis in the current pregnancy of any major birth defect prior to enrolment; and agreed to the requirements of the study, including interviews, release of medical records and the physical examination of liveborn infants. Women who were currently pregnant who had an exposure to methotrexate, chlorambucil, cyclophosphamide, adalimumab, etanercept, infliximab or any other biologic at any time in the post-conception period, or leflunomide within 1 year prior to conception, were excluded from the prospective cohort. Women in the exposure series were comprised of those who had received any dose of abatacept at any time from the first day of the LMP up to the date of delivery but did not qualify for the prospective cohort for any of the reasons previously listed, including retrospective reports where the outcome of pregnancy was already known at the time of enrolment.

### Maternal interviews

All participants completed up to three telephone interviews during pregnancy (if still pregnant) and one interview after delivery. The initial interview included questions regarding pregnancy history; current health history; pre-pregnancy height and weight; maternal and paternal occupation; race/ethnicity; education; current medication prescriptions and over-the-counter use; environmental or occupational exposures; use of caffeine, alcohol, tobacco or illicit drugs; current pregnancy complications and illnesses; and a history of onset and other characteristics of RA or PsA. Participants responded to three severity/disease impact assessment questionnaires to gather information about RA or PsA status at each interview. These included a portion of the Clinical Health Assessment Questionnaire (CLIN-HAQ) [5], i.e. the HAQ Disability Index (HAQ-DI) [6] (RA1), the global pain scale (RA2) and a question on overall impact of the disease on health (RA3). Women completed an outcome interview that collected information on the date of delivery, delivery hospital and mode of delivery; infant sex, birth measurements and Apgar scores; delivery or birth complications, pregnancy complications and neonatal complications; type and length of hospital stay for the mother and the infant; method of infant feeding; pregnancy weight gain; and any other additional exposures and results of prenatal tests since the last interview. Outcome interviews for pregnancies resulting in pregnancy loss were also conducted. These interviews collected information on the date and type of outcome, hospital location (if applicable), sex and birth size, prenatal diagnosis and/or pathology results and any additional exposures and results of prenatal tests since the last interview.

### Medical record abstraction and dysmorphological evaluation

Following the completion of the outcome interview, participants were provided medical records release forms. Signed medical records release forms and a pregnancy exposure diary form were returned by the participants. Liveborn infants of enrolled women were offered a physical examination conducted by the study’s paediatric dysmorphologists/geneticists to evaluate infants for minor structural defects. These examinations were conducted using a standard checklist of ≈130 minor structural defects and in a blinded manner with respect to exposure [7].

### Outcomes

Information on major and minor structural defects identified up to 1 year of age, and specifically evidence of a pattern in exposed infants, was the primary outcome of interest. Major birth defects were classified using the Metropolitan Atlanta Congenital Defects Program (MACDP) coding system [7]. A pattern of minor malformations was defined as the presence of at least three identical minor anomalies in two or more infants exposed to abatacept in the first trimester.

Secondary outcomes included spontaneous abortion (SAB), stillbirth, preterm delivery, birth size and postnatal growth at about 1 year of age ≤10th centile for age and sex using standard growth curves for full and preterm infants [8, 9].

### Statistical methods

Descriptive analyses were performed, as the sample size was determined to be too limited to perform statistical comparisons to an unexposed cohort. Maternal and infant characteristics and outcomes were described using numbers and percentages and means and s.d.s. All statistical analyses were performed using SAS version 9.4 (SAS Institute, Cary, NC, USA).

### Ethics approval

This study was approved by UCSD’s Institutional Review Board (approval 141162). Verbal informed consent was obtained from all participants to conduct the interviews and written informed consent was obtained from participants to conduct the physical examinations.

## Results

### Maternal characteristics

Between 2007 and 2019, 30 pregnant women were enrolled into the study. Of these, 16 were enrolled in the prospective cohort and 14 in the exposure series. The majority in both groups were White, non-Hispanic and of medium to high socio-economic status (Table 1). The majority in both groups were >30 years of age, had a normal pre-pregnancy BMI and resided in the USA. Spontaneous abortion in a previous pregnancy was similar between groups. Elective termination in a previous pregnancy was infrequent in both groups. In both groups, the majority of the pregnancies were intended. However, the prospective cohort was nominally more likely to be primiparous and less likely to have had a previous preterm delivery. Of the pregnancies included in this study, only one in the prospective cohort was conceived through *in vitro* fertilization. Comorbidity with thyroid disease was nominally higher in the exposure series; however, all other comorbidities were similar across both groups. Gestational age at enrolment was, on average, 11.4 weeks (s.d. 4.7) in the prospective cohort and later in the exposure series, with a mean of 15.1 weeks (s.d. 9.8), due to the fact that women in the exposure series could enrol after 20 weeks’ gestation or after completion of pregnancy (Table 1). Participants in both groups had abatacept exposure sometime between the LMP to the date of conception only, first trimester only or at least once later in pregnancy (Table 1). Alcohol use was similar across both groups and there was no tobacco use reported in pregnancy in either group (Table 1).

**Table:** **Table 1** Selected maternal characteristics, exposures and prenatal tests of women exposed to abatacept during pregnancy, 2007–2019.

Characteristics	Abatacept exposed (*N* = 16)	Abatacept exposure series (*N* = 14)
Maternal age at estimated due date (years), *n* (%)		
≤29	3 (18.8)	4 (28.6)
30–34	8 (50.0)	5 (35.7)
>34	5 (31.3)	5 (35.7)
Maternal age at estimated due date, years, mean (s.d.)	33.6 (4.9)	32.56 (5.0)
Maternal ethnicity category, *n* (%)		
Non-Hispanic	14 (87.5)	12 (85.7)
Hispanic	2 (12.5)	2 (14.3)
Maternal race category, *n* (%)		
White	10 (62.5)	10 (71.4)
Black	2 (12.5)	1 (7.1)
Asian/Pacific Islander	2 (12.5)	0 (0.0)
Native American	1 (6.3)	0 (0.0)
Unknown	1 (6.3)	3 (21.4)
Maternal education category (years), *n* (%)		
≤15	3 (18.8)	7 (50.0)
>15	13 (81.3)	7 (50.0)
Hollingshead socio-economic category, *n* (%)		
Low (4 or 5)	0 (0.0)	2 (14.3)
Medium–high (1–3)	15 (93.8)	11 (78.6)
Unknown	1 (6.3)	1 (7.1%)
Pre-pregnancy height, cm, mean (s.d.)	168.3 (7.9)	167.6 (7.78)
Pre-pregnancy body weight, kg, mean (s.d.)	73.4 (23.9)	73.2 (16.8)
Pre-pregnancy BMI category (kg/m^2^), *n* (%)		
<24.9 (normal weight)	13 (81.3)	7 (50.0)
25–29.9 (overweight)	0 (0.0)	5 (35.7)
≥30 (obese)	3 (18.8)	2 (14.3)
Gravidity category, *n* (%)		
1	5 (31.3)	5 (35.7)
>1	11 (68.8)	9 (64.3)
Parity category, n (%)		
0	12 (75.0)	6 (42.9)
>0	4 (25.0)	8 (57.1)
Previous spontaneous abortion category, *n* (%)		
0	10 (62.5)	9 (64.3)
>0	6 (37.5)	5 (35.7)
Previous elective termination category, *n* (%)		
0	14 (87.5)	13 (92.9)
>0	2 (12.5)	1 (7.1)
Previous preterm delivery, *n* (%)	1 (6.3)	3 (21.4)
Any previous child with a birth defect, *n* (%)	1 (6.3)	1 (7.1)
In vitro fertilisation with this pregnancy, *n* (%)	1 (6.3)	0 (0.0)
Gestational age at time of enrolment, weeks, mean (s.d.)	11.4 (4.7)	15.1 (9.8)^a^
Gestational age at time of enrolment category (weeks), *n* (%)		
≤13	11 (68.8)	4 (28.6)
13.1–19.9	5 (31.3)	2 (14.3)
≥20	0 (0.0)	8 (57.1)
Year of enrolment, *n* (%)		
2007–2009	1 (6.3)	3 (21.4)
2010–2013	3 (18.8)	6 (42.9)
2014–2016	7 (43.8)	3 (21.4)
2017–2019	5 (31.3)	2 (14.3)
Referral source, *n* (%)		
Sponsor, healthcare professional	10 (62.5)	10 (71.4)
Internet, patient support group or other	5 (31.3)	4 (28.6)
OTIS member service	1 (6.3)	0 (0.0)
Geographic area of residence, *n* (%)		
USA	13 (81.3)	12 (85.7)
Canada	2 (12.5)	2 (14.3)
Unknown	1 (6.3)	0 (0.0)
Maternal comorbidities, *n* (%)		
Thyroid disease	3 (18.8)	6 (42.9)
Asthma	1 (6.3)	1 (7.1)
Other autoimmune disease	0 (0.0)	1 (7.1)
Pre-gestational hypertension	1 (6.3)	2 (14.3)
Maternal complications, *n* (%)		
Pregnancy-induced hypertension [among pregnancies with livebirth(s)]	0 (0.0)	0 (0.0)
Pre-eclampsia [among pregnancies with livebirth(s)]	0 (0.0)	3 (21.4)
Gestational diabetes [among pregnancies with livebirth(s)]	1 (3.3)	0 (0.0)
Intended pregnancy, *n* (%)	13 (81.3)	11 (78.6)
Years since diagnosis of primary disease (RA or PsA), mean (s.d.)^b^^,c^	7.9 (5.1)	7.9 (4.2)
Number of autoimmune diagnoses, *n* (%)		
1	0 (0.0)	2 (14.3)
>1	15 (93.8)	7 (50.0)
Disease severity measures by timing of administration in pregnancy, mean (s.d.)^d^		
Enrolment (abatacept exposed, *n* = 16; abatacept exposure series, *n* = 12)		
RA 1	0.5 (0.7)	0.8 (1.0)
RA 2	19.3 (22.2)	29.5 (36.4)
RA 3	29.2 (28.9)	24.8 (33.2)
Second trimester (abatacept exposed, *n* = 11; abatacept exposure series, *n* = 3)		
RA 1	0.3 (0.4)	0.5 (0.7)
RA 2	19.3 (21.0)	36.7 (40.4)
RA 3	21.1 (24.8)	33.3 (49.3)
Third trimester (abatacept exposed, *n* = 12; abatacept exposure series, *n* = 6)		
RA 1	0.5 (0.6)	0.7 (0.7)
RA 2	17.3 (22.1)	41.8 (27.6)
RA 3	19.8 (25.7)	41.7 (37.6)
Gestational timing of exposure to abatacept, *n* (%)		
Data available	15 (93.8)	14 (100.0)
Exposure prior to the LMP	0	0
LMP to less than the date of conception only	1 (6.7)	4 (28.6)
First trimester only	7 (46.7)	7 (50.0)
First and second trimesters only	0	0
First and third trimesters only	0	0
First, second and third trimesters	7 (46.7)	3 (21.4)
Second trimester only	0	0
Second and third trimesters only	0	0
Third trimester only	0	0
Prednisone and/or systemic oral corticosteroid, *n* (%)	3 (18.8)	6 (42.9)
Gestational timing of exposure to prednisone and/or systemic oral corticosteroid, *n* (%)		
LMP to less than the date of conception only	0 (0.0)	0 (0.0)
First trimester only	1 (33.3)	0 (0.0)
First and second trimesters only	0 (0.0)	0 (0.0)
First, second and third trimesters	0 (0.0)	1 (16.7)
Second trimester only	0 (0.0)	0 (0.0)
Second and third trimesters only	0 (0.0)	2 (33.3)
Third trimester only	2 (66.7)	3 (50.0)
No exposure from the LMP to the end of the third trimester	0 (0.0)	0 (0.0)
Dose of prednisone and/or systemic oral corticosteroid per treated week, mg/week, mean (s.d.)	56.7 (18.9)	60.5 (28.6)
Total number of weeks exposed to prednisone and/or systemic oral corticosteroid, *n* (%)		
Unexposed or LMP to date of conception only	0 (0.0)	0 (0.0)
0.1–4	1 (33.3)	0 (0.0)
4.1–12	0 (0.0)	0 (0.0)
>12	2 (66.7)	6 (100.0)
Influenza vaccine in pregnancy, *n* (%)	10 (62.5)	8 (57.1)
Prenatal multivitamin or folic acid supplements, *n* (%)		
Began prior to conception	11 (68.8)	7 (50.0)
Post-conception only	5 (31.3)	7 (50.0)
Alcohol use in pregnancy (post-conception), *n* (%)	4 (25.0)	3 (21.4)
Tobacco use in pregnancy (post-conception), *n* (%)	0 (0.0)	0 (0.0)
Human teratogens (post-conception), *n* (%)	0 (0.0)	1 (7.1)
Prenatal diagnostic tests performed prior to enrolment, *n* (%)		
Ultrasound Level 1	10 (62.5)	14 (100.0)
Ultrasound Level 2	2 (12.5)	7 (50.0)
Chorionic villus sampling (CVS)	0 (0.0)	0 (0.0)
Amniocentesis	0 (0.0)	1 (7.1)
Prenatal diagnostic tests performed anytime in pregnancy (post-conception), *n* (%)		
Ultrasound level 1	16 (100.0)	14 (100.0)
Ultrasound level 2	13 (81.2)	12 (85.7)
Chorionic villus sampling	0 (0.0)	0 (0.0)
Amniocentesis	0 (0.0)	2 (14.3)

a Data shown are for pregnancies in the exposure series that enrolled during pregnancy.

b Among the abatacept exposed, 14 pregnancies had a primary diagnosis of RA and 2 pregnancies had a primary diagnosis of PsA.

c Among the exposure series, 8 pregnancies had a primary diagnosis of RA and 1 pregnancy in the exposure series had a primary diagnosis of PsA; data unavailable for primary diagnosis for 5 pregnancies. Women who were currently pregnant and had exposure to methotrexate, chlorambucil, cyclophosphamide, adalimumab, etanercept, infliximab or any other biologic at any time in the post-conception period, or leflunomide within 1 year prior to conception, were excluded from the prospective cohort.

d RA1 refers to the severity assessment questionnaire from the CLIN-HAQ/HAQ-DI [5, 6] and participants respond using the following scale: without any difficulty, 0; with some difficulty, 1; with much difficulty, 2; unable, 3. Participants are given a score between 0 and 3. RA2 refers to the global pain scale and participants are asked to choose a number between 0 and 100 that best describes the severity of the pain in the past week. A score of 0 indicates no pain and 100 is severe pain. RA3 refers to the overall impact of the disease on health, where participants are asked to consider all the ways the illness affects them and rate how they are doing overall. A score of 0 indicates the participant is doing very well and a score of 100 is very poor.

For the CLIN-HAQ/HAQ-DI (RA 1) and global pain scale (RA 2), participants in the prospective cohort had lower scores than the exposure series and higher scores for the overall impact of the disease on their health (RA 3) (Table 1). During the second trimester, 11 from the prospective cohort and 3 from the exposure series completed the questionnaires. Across all three questionnaires, participants in the prospective cohort had lower scores compared with the exposure series (Table 1). During the third trimester, 12 from the prospective cohort and 6 from the exposure series completed the questionnaires. Participants in the prospective cohort scored lower on all three questionnaires compared with the exposure series (Table 1).

### Exposures

In both groups, the majority of the participants were exposed to at least one dose of abatacept only in the period from the LMP to end of the first trimester (53.4% in the prospective cohort and 78.6% in the exposure series) and the remainder were exposed throughout pregnancy. A higher proportion of women in the exposure series were also treated with systemic steroids in pregnancy (42.9%) than in the prospective cohort (18.8%) (Table 1).

### Pregnancy and infant outcomes

In the prospective cohort, 13/16 (81.3%) pregnancies resulted in at least one live birth. Two pregnancies of 13 ending in a livebirth (15.4%) involved a child with a major birth defect; one infant had congenital chordee and one had a haemangioma, which met MACDP criteria due to size. Two of 16 pregnancies (12.5%) ended in SAB; 2/13 livebirths (15.4%) were delivered preterm and 1/16 (6.3%) was lost to follow-up (Table 2 and Supplementary Table S1).

**Table:** **Table 2** Outcomes in pregnancies exposed to abatacept (*n* = 30).

Outcomes	Abatacept exposed (*N* = 16), *n* (%)	Abatacept exposure series (*N* = 14), *n* (%)
Livebirth	13 (81.3)	14 (100.0)
Major birth defects in pregnancies ending with live-born infants	2/13^a^ (15.4)	5/14 (35.7)
Major birth defects in all pregnancies excluding lost to follow-up	2/15 (13.3)	5/14 (35.7)
Dysmorphology examination	6/13 (46.2)	7/13 (53.8)
Pattern of minor birth defects in infants who received the physical examination	0/6 (0.0)	0/7 (0.0)
Spontaneous abortion	2/16 (12.5)	0/14 (0.0)
Stillbirth	0/16 (0.0)	0/14 (0.0)
Termination	0/16 (0.0)	0/14 (0.0)
Preterm delivery	2/13 (15.4)	6/14 (42.9)
Small for gestational age ≤10th centile on weight	1/13 (7.7)	2/14 (14.3)
Small for gestational age ≤10th centile on length	0/13 (0.0)	0/13 (0.0)
Small for gestational age ≤10th centile on head circumference	2/11 (18.2)	0/12 (0.0)
Postnatal growth ≤10th centile on weight at 1 year of age	0/16 (0.0)	2/14 (14.3)
Postnatal growth ≤10th centile on length at 1 year of age	0/16 (0.0)	2/14 (14.3)
Postnatal growth ≤10th centile on head circumference at 1 year of age	0/16 (0.0)	2/14 (14.3)
Lost to follow-up	1/16 (6.3)	0/14 (0.0)

a One pregnancy involved twins ending in one live birth and one spontaneous abortion; counted only in the outcome of livebirth.

In the exposure series group, 14/14 pregnancies (100.0%) resulted in at least one live birth. Three of the 14 livebirths (21.4%) had a major birth defect (pyloric stenosis, cleft lip and palate, and patent foramen ovale persisting to at least 6 weeks of age). An additional two pregnancies in the exposure series (14.3%) had a major birth defect in association with a genetic or chromosomal alteration that were unlikely to be related to exposure. Six of 14 pregnancies (42.9%) were delivered preterm. There were no SABs reported and no enrolled pregnancies lost to follow-up (Table 2 and Supplementary Table S1). No pattern of major birth defects was evident in either the cohort or the exposure series (Table 2 and Supplementary Table S1).

A paediatric dysmorphology examination for minor birth defects was completed for six infants in the prospective cohort and seven in the exposure series group. No patterns of minor birth defects were identified in either group.

The number of liveborn infants who were small for gestational age (≤10th centile for sex and gestational age at birth) or smaller (≤10th centile at about 1 year of age) for weight, length or head circumference appeared to be higher than the expected range of 10% in both groups, based on very small numbers (Table 2).

## Discussion

In this study, the incidence/birth prevalence of selected maternal and infant outcomes in 30 abatacept-exposed pregnancies was examined. Although the number of exposed pregnancies was small, there were no patterns of major or minor anomalies identified in either group. Higher rates of major birth defects and preterm delivery were seen in the exposure series. However, the biases inherent among those enrolled who did not meet the prospective cohort criteria, including those enrolled retrospectively, may explain these differential findings. Furthermore, this study observed seven major birth defects in 27 pregnancies ending with a liveborn infant, which is higher than the 3–5% expected rate in the general population [10]. This may be due in part to the methods of ascertainment of major birth defects in this study. First, infants were routinely followed to 1 year of age, where defects not readily identified at birth could be diagnosed. In addition, data on major birth defects were collected through that time period from a comprehensive set of sources, including maternal report, medical records and the dysmorphology exam. Finally, in the exposure series, where prospective ascertainment was not required, it is expected that adverse outcomes including major birth defects will be differentially more likely to be reported. It should also be recognized that the stability of the estimates of the birth prevalence of major birth defects was limited by the small sample size and thus the number of birth defects identified could be due to chance.

There are currently few studies in the literature documenting the safety and use of abatacept in pregnancy. The findings of this study are consistent with a prior descriptive analysis published by Kumar *et al.* [11] of 161 pregnancies exposed to abatacept reported to the manufacturer using pharmacovigilance data from clinical trials, registries and spontaneous post-marketing reports. This analysis of pharmacovigilance data did not detect any patterns of congenital anomalies or evidence of an increased risk of SAB. In the OTIS Registry study, there were two SABs in the prospective cohort (12.5%), a rate that is similar to the 15–20% expected in the general population [12]. It is important to note that there is some overlap prior to 2015 between this study and the Kumar *et al.* [11] publication, which included OTIS MTB pregnancies in their analysis.

Based on the 2020 ACR Guideline for the Management of Reproductive Health in Rheumatic and Musculoskeletal Diseases, the ACR recommends to conditionally continue the use of abatacept while trying to conceive but to discontinue once the pregnancy is confirmed [13].

There are limitations and strengths to this study. As in any volunteer registry, there is the potential for selection bias, which may limit external validity. In addition, the sample size was too small to perform statistical comparisons to any reference group. Strengths of the study are low lost-to-follow-up rates and the extensive capture of exposure, outcome and covariate data from maternal reports and medical records abstraction. Furthermore, the subset of liveborn infants who received a dysmorphology examination provided some reassurance that no specific pattern of minor anomalies was identified.

## Conclusion

While this study observed a higher frequency of major birth defects than the general population, which could be due in part to methods of ascertainment, importantly no patterns of major or minor birth defects were identified. Selected adverse outcomes were more frequent in the exposure series, likely due to biases inherent in the exclusion criteria for the prospective cohort. Future studies with a larger sample size are needed.

## Supplementary Material

rkag084_Supplementary_Data

## Data Availability

Data availability can be discussed with the corresponding author.
