# Supplementary material for: Pregnancy and infant outcomes following abatacept exposure during pregnancy
Source: Rheumatol Adv Pract. 2026 Jul 20;10(3):rkag084. doi: 10.1093/rap/rkag084 (PMC13451083; doi:10.1093/rap/rkag084)
Supplement: Supplementary file 1 [file rkag084_supplementary_data.docx]

**Supplementary Table S1. Major Birth Defects in Infants Exposed to Abatacept**

**Major Birth Defects in Prospective Cohort**

1. Congenital chordee

2. Hemangioma, skin and subcutaneous, NOS

**Major Birth Defects in the Exposure Series**

1. Congenital hypertrophic pyloric stenosis

2. Cleft lip with cleft palate

3. Patent foramen ovale

**Major Birth Defects of Chromosomal or Genetic Etiology in the Exposure Series**

1. Balanced chromosome translocation (46XX, balanced translocation between 14/15) with microcephaly, persistent with right aortic arch, and pulmonary atresia with ventricular septal defect

2. 14Q 11.2 microdeletion with anomalous optic nerves (bilateral) with vascular abnormality and developmental delay
